# The Gene Signature Associated with Hepatocellular Carcinoma in Patients with Nonalcoholic Fatty Liver Disease

**DOI:** 10.1155/2021/6630535

**Published:** 2021-04-02

**Authors:** Jiayun Ge, Yannan Bai, Bo Tang, Dong Wei, Maolin Yan

**Affiliations:** ^1^Department of Hepatobiliary Surgery, The Second Affiliated Hospital, Kunming Medical University, 374 Dianmian Road, Wuhua District, Kunming, Yunnan (650101), China; ^2^Department of Hepatobiliary Surgery, Fujian Provincial Hospital, The Shengli Clinical Medical College of Fujian Medical University, Fuzhou (350001), Fujian, China

## Abstract

**Background:**

Nonalcoholic fatty liver disease (NAFLD) is becoming a critical risk of hepatocellular carcinoma (HCC). As both NAFLD and HCC are heterogeneous diseases, this study aims to identify the similarity between the subtypes of NAFLD and HCC based on gene modules.

**Methods:**

Coexpressed gene modules were extracted for both NAFLD and HCC. The association between the coexpressed gene modules of NAFLD and HCC was evaluated by Fisher's exact test. The overlapping coexpressed gene module was validated in three independent human NAFLD datasets. Furthermore, the preserved gene module was assessed in four independent NAFLD mouse datasets. The significantly enriched motifs within the gene module were inferred from upstream sequences.

**Results:**

Four coexpressed gene modules were extracted from NAFLD. Of the four coexpressed gene modules, one was significantly overlapping with a module of HCC. This overlapping gene module was regarded as the HCC-associated NAFLD gene module (HANM). Enrichment analysis of biological processes revealed inflammatory response in HANM. Specifically, within the inflammatory response biological process, IL-17, TNF-*α*, and NF-*κ*B signaling pathways were enriched. HANM was found to be strongly or moderately conserved across four mouse NAFLD datasets. Motif analysis of the upstream genomic sequences of HANM revealed nine transcription factors (FLI1, NRF1, ZBTB33, ELK1, YY1, ZNF143, TAF1, SF1, and E2F1), of which three transcription factors (YY1, E2F1, and ZNF143) were significantly highly expressed in the NAFLD patients and exhibited survival significance in HCC.

**Conclusion:**

This study demonstrated a robust way to identify the sharing gene signature between subtypes of NAFLD and HCC, which contributed to a comprehensive understanding of pathogenesis from NAFLD to HCC.

## 1. Background

Nonalcoholic fatty liver disease (NAFLD) is a spectrum of chronic liver disease spanning excessive cytoplasmic retention of triglyceride, steatosis, nonalcoholic steatohepatitis (NASH), and hepatic fibrosis and cirrhosis [1]. With the global pandemic of obesity, NAFLD is becoming a critical issue [2]. The prevalence of NAFLD increased from 15% to 25% during 2005–2010 [3]. Of NAFLD patients, approximately 2.4–12.8% will develop into progressive liver disease [4] including hepatocellular carcinoma (HCC). However, NAFLD-associated HCC risk has been largely underestimated because of misdiagnosis [5]. Recent studies have recognized NAFLD as the most common risk for HCC [6]. A USA cohort study has reported that 59% of HCC patients were associated with NAFLD or NASH [7]. Other studies from Germany [8], Italy [9], and Japan [10] have reported that 41.7–49% of HCC patients without cirrhotic background were related to NAFLD. Thus, it is urgent to understand the associated mechanisms behind them.

NAFLD is a disease associated with the necroinflammatory process [11], DNA damage response [12], immune responses [13], and oxidative stress (reactive oxygen species, oxidation, and endoplasmic reticulum) [14]. However, both NAFLD and HCC are highly heterogeneous. Direct comparison between them with regular statistical tests could be problematic. Alternatively, weighted correlation network analysis (WGCNA) can subtype HCC and NAFLD patients and identify the underlying coexpressed gene modules at the same time [15]. The underlying coexpressed gene module of the associated HCC and NAFLD subtypes, hereafter, was named HANM (the HCC-associated NAFLD module). The construction of HANM has multiple applications. First, it could improve our understanding of the NAFLD-to-HCC conversion mechanism. Second, the identification of HANM could help to identify the patients with HCC risk. Third, HANM could be used to assess the animal models which were used in NAFLD/HCC research studies [16–19].

In this study, we first identified the coexpressed gene modules of both NAFLD and HCC. Module overlapping analysis was used to find HANM. HANM was then validated in three independent NAFLD human datasets and four mouse datasets. Functions of HANM were further characterized with gene ontology, KEGG, and motif analysis.

## 2. Materials and Methods

### 2.1. Datasets

Human datasets (GSE89632, GSE59045, GSE126848, and GSE83452) and mouse datasets (GSE137449, GSE128940, GSE114261, and GSE83596) were obtained from the Gene Expression Omnibus (GEO) database (http://www.ncbi.nlm.nih.gov/geo/). The raw data were called and normalized with the Robust Multiarray Average (RMA) method and log2-transformed. The other HCC gene expression dataset was downloaded from TCGA (https://portal.gdc.cancer.gov/) and used in accordance with the publication guidelines of TCGA (http://cancergenome.nih.gov/publications/publicationguidelines). The value of fragments per kilobase million was used as gene expression.

### 2.2. Coexpressed Gene Module Construction

*R* package WGCNA [15] was used to construct the coexpressed gene modules according to its manual (https://horvath.genetics.ucla.edu). The parameter, soft power, was chosen by a scale-free topology model fit. An unsigned correlation coefficient is regarded as the distance between two samples. A topological network was thus constructed, hierarchically clustered, and dynamically cut into multiple gene modules. Gene modules were merged according to Pearson's correlation coefficient between their eigengenes.

### 2.3. Gene Module Preservation Analysis

To evaluate the clinical significance of coexpressed gene modules, the overall survival proportion was regressed against survival time with the Cox proportional hazard model. Expression over the median was defined as the high expression and that below the median as low expression. The Cox proportional hazard model was built with the *R* package “survival.” A log-likelihood test was used to assess the significance. Modules with a higher survival probability were further enriched with gene ontology biological processes.

Human genes were mapped to mouse genes (orthologous genes) according to the Mouse Genome Informatics database (http://www.informatics.jax.org). Gene module preservation was estimated by averaging the several preservation statistics generated through 1000 permutations of the original data. A *Z*_summary_ value is calculated, which summarizes the evidence that a module is preserved and indicative of module robustness and reproducibility. According to WGCNA recommendation [20], the preservation was defined as strong for *Z*_summary_ > 10, moderate for 2 < *Z*_summary_ <10, and weak for *Z*_summary_<2.

### 2.4. Gene Function Analysis

ClusterProfiler [21] was utilized to perform the enrichment analysis of biological processes of gene ontology and KEGG pathways (https://www.genome.jp).

### 2.5. Searching Shared Transcription Factors

iRegulon (http://iregulon.aertslab.org) [22] was used to perform the enrichment analysis of shared transcription factors in a gene module. As suggested, the transcription factors with a normalized enrichment score >3.0 were considered significant.

### 2.6. Survival Analysis

The survival analysis was conducted with a web service [23]. The parameter cutoff of gene expression was set as “optimal.” Risk factors (alcohol consumption and hepatitis virus) were set none. Totally, 91 samples were used in this analysis.

## 3. Results

### 3.1. Coexpressed Gene Modules in NAFLD Patients

**Figure 1:**
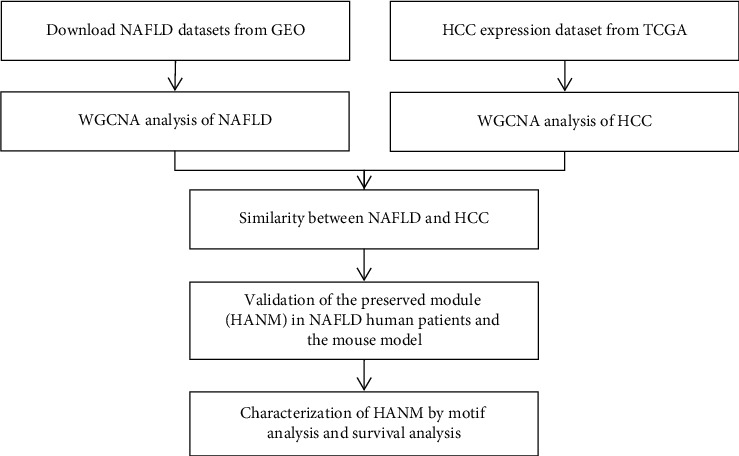
Flowchart of this study. Validation of the preserved module (HANM) in NAFLD human patients and the mouse model.

**Figure 2:**
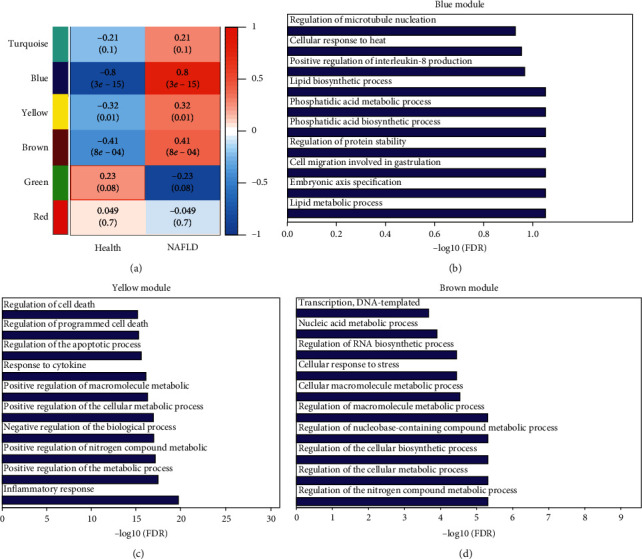
WGCNA analysis of expression profiles from healthy and NAFLD samples. (a) The association between the gene modules and two NAFLD statuses (health and NAFLD) is indicated by Pearson's correlation coefficient and *p* value (in parenthesis). Blue, yellow, and brown modules are significantly highly expressed in NAFLD in comparison to healthy samples. (b) The significance of biological process enrichment for the blue gene module is indicated by the negative log10-transformed false discovery rate (FDR). (c) The significance of biological process enrichment for the yellow gene module is indicated by −log10 (FDR). (d) The significance of biological process enrichment for the brown gene module is indicated by −log10 (FDR).

The whole analysis procedure is illustrated in Figure 1. First, gene expression of a NAFLD dataset, GSE89632, was directly parsed from the prepared series matrix file and hierarchically clustered. Dynamic cutting and merging of the hierarchical clustering tree generated six coexpressed gene modules. They were named with different colors automatically by the WGCNA program (Supplementary Figure 1). Module similarity was estimated by Pearson's correlation coefficient between module eigengenes. Modules with a similarity >0.75 were merged. Pearson's correlation coefficient between the eigengenes of each merged module and the NAFLD status is shown in Figure 2(a). Three gene modules (blue, yellow, and brown) were significantly associated with NAFLD. Gene ontology analysis of biological processes found no enriched biological process in the blue module (Figure 2(b)). The yellow module was found enriched in the inflammatory response, positive regulation of the metabolic process, positive regulation of the nitrogen compound metabolic process, and regulation of the apoptotic process (Figure 2(c), Supplementary Table 1). Specifically, of the inflammatory response biological process, IL-17, TNF-*α*, and NF-*κ*B signaling pathways were enriched (Supplementary Table 2). The brown gene module is found enriched in the regulation of the nitrogen compound metabolic process, regulation of the cellular biosynthetic process, and cellular response to stress (Figure 2(d)).

### 3.2. HCC-Associated NAFLD Coexpressed Gene Modules

**Figure 3:**
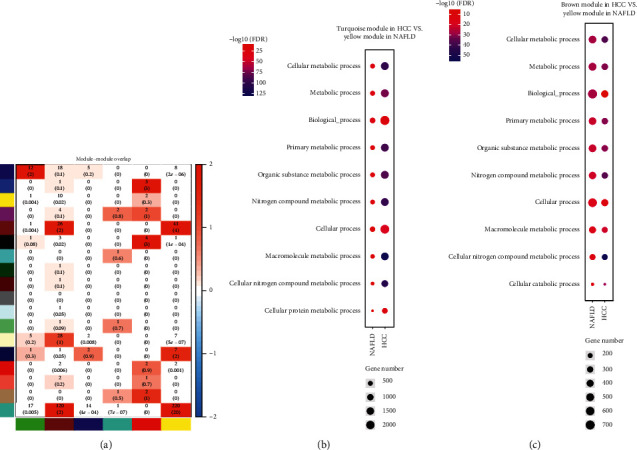
Module overlap between human NAFLD and HCC samples. (a) Module overlap is indicated by the number of shared genes and the negative log10-transformed *p* value (in parenthesis). (b) Comparison of biological processes between the yellow module of human NAFLD and turquoise module of human HCC. (c) Comparison of biological processes between the yellow module of human NAFLD and brown module of human HCC.

An HCC gene expression dataset was downloaded from TCGA (https://portal.gdc.cancer.gov/). Gene modules in HCC were constructed as NAFLD (Supplementary Figure 2). After dynamical cutting of the coexpressed hierarchical tree, we identified 18 gene modules in HCC. These gene modules were then compared to the modules from the NAFLD dataset. Differential significance was assessed by Fisher's exact test (Figure 3(a)). Of the six gene modules from the NAFLD dataset, the yellow gene module was significantly associated with the gene modules (turquoise and brown) from HCC (*p* value <0.001). The two gene modules from HCC were functionally similar to the yellow module of NAFLD in the biological processes (Figures 3(b) and 3(c)), molecular functions (Supplementary Figures 3A-3B), and cellular components (Supplementary Figures 3C-3D).

### 3.3. Gene Module Consistency in Human Patients and Mouse Models with NAFLD

**Figure 4:**
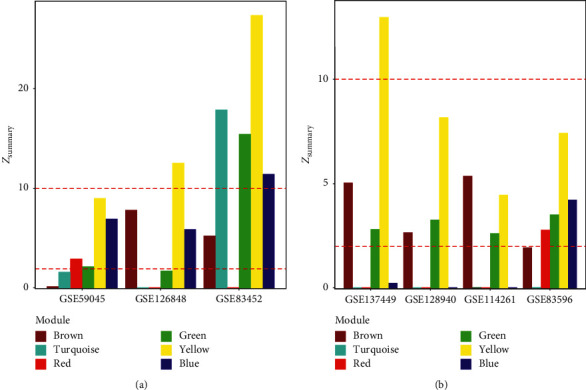
Gene module preservation between human and mouse. (a) The gene module preservation of the human NAFLD gene modules in human NAFLD. (b) The gene module preservation of the human NAFLD gene modules in mouse NAFLD models.

The identified gene modules associated with HCC were further validated in three independent human NAFLD datasets. The three datasets (GSE59045, GSE126848, and GSE83452) were downloaded from the GEO database. A permutation test was used to calculate the empirical *p* value of the statistics *Z*_summary_, which indicated the preservation of the coexpressed gene network in one dataset against another. The preservation was defined as strong for *Z*_summary_ > 10, moderate for 2 < *Z*_summary_ < 10, and weak for *Z*_summary_ < 2. The yellow gene module was found strongly preserved in two datasets and moderately conserved in the other one (Figure 4(a)).

We further validate these gene modules in mouse models. Four datasets (GSE137449, GSE128940, GSE114261, and GSE83596) from mouse models were compiled. *Z*_summary_ scores indicated that the yellow module was the most conserved gene module between NAFLD human patients and mouse models. The yellow module was strongly conserved in GSE137449, moderately conserved in GSE128940 and GSE83596, and weakly conserved in GSE114261 (Figure 4(b)). The yellow module was, hereafter, named HANM (the HCC-associated NAFLD module, Supplementary Table 1).

### 3.4. Motif Characterization of HANM

**Figure 5:**
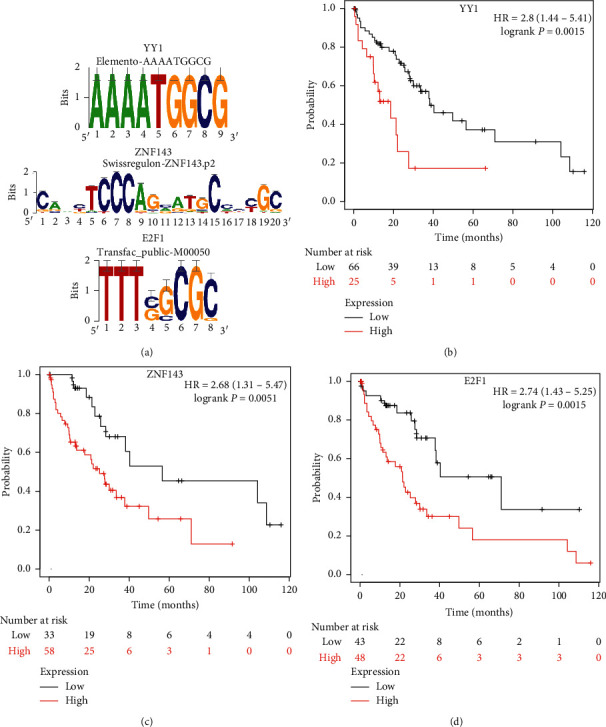
Transcription factors regulating the HCC-associated NAFLD module. (a) The enriched motifs of the HCC-associated NAFLD module. (b) The Kaplan–Meier plot for YY1 in human liver cancer. (c) The Kaplan–Meier plot for ZNF143 in human liver cancer. (d) The Kaplan–Meier plot for E2F1 in human liver cancer.

To examine the characters of HANM, we studied the enrichment of its upstream motifs through a web service, iRegulon (http://iregulon.aertslab.org) [22]. The enriched motifs are shown in Figure 5(a). Those motifs were bound by the transcription factors including FLI1, NRF1, ZBTB33, ELK1, YY1, ZNF143, TAF1, SF1, and E2F1 (Table 1). Likewise, we also enriched the upstream motifs of HANM homolog genes in mice. The enriched motifs were bound by the transcription factors including ZBTB33, NRF1, FLI1, ZFP143, UQCRB, ZFP42, E2F3, and RARA (Table 2). Three transcription factors (FLI1, NRF1, and ZBTB33) were shared by the two species.

We selected the samples with high correlation to the eigengene of HANM (Pearson's correlation coefficient>0.9, *p* < 0.05). These samples were then compared to healthy samples by each HANM gene with the limma package (https://bioconductor.org). Fold change >1.8 and false discovery rate (FDR) < 0.05 were used as a cutoff. Totally, 131 differentially expressed genes were found. Of the nine transcription factors, six (ZBTB33, YY1, TAF1, E2F1, SF1, and ZNF143) were significantly highly expressed in the NAFLD patients with FDR < 0.05 and fold change > 1.8.

We further investigated the survival effect of these six differentially expressed transcription factors. Patients were split into two groups by the optimal cutoff of each gene [23]. A log-rank test was used to assess the survival differences between the two groups. Of the six differentially expressed transcription factors, YY1, ZNF143, and E2F1 were significantly associated with survival with *p* value = 0.0015, 0.0051, and 0.0015, respectively. Their Kaplan–Meier curves are plotted in Figures 5(b)–5(d).

## 4. Discussion

In this study, we had inferred multiple coexpressed gene modules in NAFLD and HCC patients. Association analysis between NAFLD and HCC identified three overlapping gene modules (blue, yellow, and brown gene modules). Only in a part of NAFLD patients, the gene expression was positively correlated with the eigengene of HANM, which was reasonable since not all HCC patients came from NAFLD. In this sense, direct NAFLD vs. HCC comparison was defective to identify the faithfully associated genes between NAFLD and HCC.

Comparing the gene modules of NAFLD to those of HCC, the brown and yellow gene modules of NAFLD were found associated with the turquoise and brown modules of HCC, respectively. Though the brown and yellow gene modules were overlapped in NAFLD and HCC, only the yellow gene module showed consistency in the independent NAFLD human and mouse datasets. Thus, we only considered the yellow gene module as HANM. Besides, HANM has the highest *Z*_summary_ score in the choline-deficient, L-amino acid-defined high-fat diet mouse model (GSE137449), which implies the usefulness of this mouse model in the study of pathogenesis from NAFLD to HCC.

HANM was enriched in immune and metabolism-related biological processes including inflammatory response, positive regulation of the metabolic process, positive regulation of the nitrogen compound metabolic process, and regulation of the apoptotic process. These biological processes had a high association with NAFLD-related HCC. Specifically, of the inflammatory response biological process, IL-17, TNF-*α*, NF-*κ*B signaling pathways were enriched (Supplementary Table 2). A mechanism study demonstrated that TNF-alpha and IL-6 can enhance HCC in NAFLD or obesity population [24], which could be activated by the elevated ER stress in NAFLD patients [25].

To further characterize HANM, we investigated the upstream transcription factors in accord to the enriched motifs upstream of HANM genes. Nine transcription factors were retrieved. Of the nine transcription factors, three transcription factors (FLI1, NRF1, and ZBTB33) were conserved in the mouse models. The function of FLI1 and ZBTB33 in NAFLD is still unclear. FLI1 is a protooncogene and can promote metastasis by regulating MMP2 signaling [26]. ZBTB33 is also found to be associated with diabetes mellitus and hepatocellular carcinoma [27]. NRF1 is clearly a NAFLD pathogenic gene. As reported, NRF1 is involved in mediating the activation of oxidative stress response genes [28]. After liver-specific deletion of NRF1, mice could develop all NAFLD features including steatosis, fibrosis, cirrhosis, and liver cancer [29, 30].

Of the nine transcription factors, six (ZBTB33, YY1, TAF1, E2F1, SF1, and ZNF143) were significantly highly expressed in the NAFLD patients with FDR < 0.05. YY1, E2F1, and ZNF143 also have survival significance in HCC. The three genes have a strong association with NAFLD according to the literature. It has been reported that YY1 is associated with NAFLD progression undergoing bariatric surgery [31]. The expression level of YY1 is significantly correlated with NAFLD biomarkers including serum glucose, insulin, HDL, LAT, and AST. E2F1 was considered a novel regulator of metabolism [32]. E2F1 deletion can completely abrogate hepatic steatosis in different murine models [33]. ZNF143 in NAFLD has been identified in an independent study [34]. ZNF143 plays a key role in the regulation of the metabolic network about cell survival and differentiation [35, 36]. These results demonstrated the reliability of the identified gene module, HANM.

It was noteworthy that NAFLD would express higher alcohol-metabolizing genes including *ADH, ALDH, CYP2E1*, and *CAT* [37]. But we failed to find any of those genes in HANM, which suggested that the alcohol-metabolizing genes, as characteristics of NAFLD, could not be the cause of further development of HCC.

In summary, this study identified an HCC-associated NAFLD module. It was then validated in multiple datasets from human and mouse models. Its functions were characterized by gene ontology, KEGG pathway, motif, and survival analysis. The identification of this HCC-associated NAFLD module could help to understand the pathogenesis from NAFLD to HCC.

## Figures and Tables

**Table 1:** Enriched transcription factors in human.

TF	NES	#Targets	#Motifs/Tracks
FLI1	5.356	373	84
NRF1	4.853	247	10
ZBTB33	4.559	114	5
ELK1	4.437	246	4
YY1	3.896	121	12
ZNF143	3.858	47	2
TAF1	3.138	405	2
SF1	3.114	115	1
E2F1	3.010	29	1

**Table 2:** Enriched transcription factors in mouse.

TF	NES	#Targets	#Motifs/Tracks
ZBTB33	5.367	152	5
NRF1	5.089	253	12
FLI1	5.006	520	86
ZFP143	4.139	110	4
UQCRB	3.713	114	2
ZFP42	3.392	71	4
E2F3	3.166	43	2
RARA	3.101	122	1

## Data Availability

The data used to support the findings of this study are available at NCBI GEO, accession numbers: GSE89632, GSE59045, GSE126848, GSE83452, GSE137449, GSE128940, GSE114261, and GSE83596.

## Supplementary Materials

Supplementary Materials Supplementary Table 1. The top 10 enriched biological processes of HANM. Supplementary Table 2. The enriched signaling pathways in the inflammatory response biological process. Supplementary Figure 1. WGCNA analysis of expression profiles of human NAFLD and healthy samples. Gene expression of human NAFLD and healthy samples was hierarchically clustered and dynamically cut into gene modules named by different colors. Gene modules with highly correlated eigengenes (Pearson's correlation coefficient > 0.75) were merged. Supplementary Figure 2. WGCNA analysis of expression profiles of human HCC samples. Gene expression of human HCC samples was hierarchically clustered and dynamically cut into gene modules named by different colors. Gene modules with highly correlated eigengenes (Pearson's correlation coefficient > 0.75) were merged. Supplementary Figure 3. Functional association between NAFLD and HCC. (a) Molecular function similarity between the turquoise module in HCC and the yellow module in NAFLD. (b) Molecular function similarity between the brown module in HCC and the yellow module in NAFLD. (c) The cellular component similarity between the turquoise module in HCC and the yellow module in NAFLD. (d) The cellular component similarity between the brown module in HCC and the yellow module in NAFLD.
