# Supplementary material for: The Gene Signature Associated with Hepatocellular Carcinoma in Patients with Nonalcoholic Fatty Liver Disease
Source: J Oncol. 2021 Apr 2;2021:6630535. doi: 10.1155/2021/6630535 (PMC8035011; doi:10.1155/2021/6630535)
Supplement: Supplementary Materials — Supplementary Table 1. The top 10 enriched biological processes of HANM. Supplementary Table 2. The enriched signaling pathways in the inflammatory response biological process. Supplementary Figure 1. WGCNA analysis of expression profiles of human NAFLD and healthy samples. Gene expression of human NAFLD and healthy samples was hierarchically clustered and dynamically cut into gene modules named by different colors. Gene modules with highly correlated eigengenes (Pearson's correlation coefficient > 0.75) were merged. Supplementary Figure 2. WGCNA analysis of expression profiles of human HCC samples. Gene expression of human HCC samples was hierarchically clustered and dynamically cut into gene modules named by different colors. Gene modules with highly correlated eigengenes (Pearson's correlation coefficient > 0.75) were merged. Supplementary Figure 3. Functional association between NAFLD and HCC. (a) Molecular function similarity between the turquoise module in HCC and the yellow module in NAFLD. (b) Molecular function similarity between the brown module in HCC and the yellow module in NAFLD. (c) The cellular component similarity between the turquoise module in HCC and the yellow module in NAFLD. (d) The cellular component similarity between the brown module in HCC and the yellow module in NAFLD. [file 6630535.f1.zip › 6630535.f1/Figure3S.pdf]

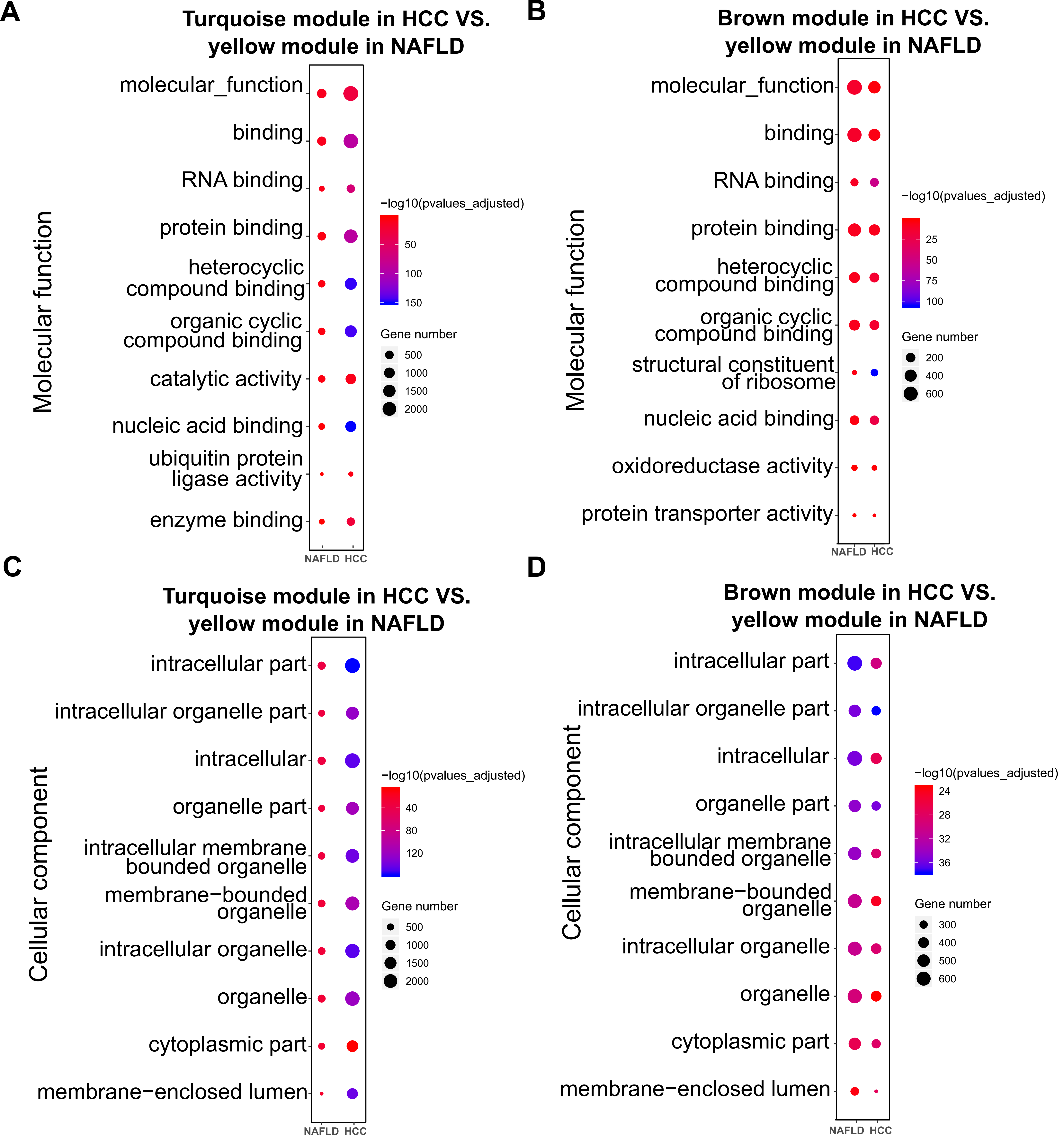
A
Turquoise module in HCC VS. yellow module in NAFLD
Molecular function
molecular_function
binding
RNA binding
protein binding
heterocyclic compound binding
organic cyclic compound binding
catalytic activity
nucleic acid binding
ubiquitin protein ligase activity
enzyme binding
NAFLD
HCC
−log10(pvalues_adjusted)
50
100
150
Gene number
500
1000
1500
2000
B
Brown module in HCC VS. yellow module in NAFLD
Molecular function
molecular_function
binding
RNA binding
protein binding
heterocyclic compound binding
organic cyclic compound binding
structural constituent of ribosome
nucleic acid binding
oxidoreductase activity
protein transporter activity
NAFLD
HCC
−log10(pvalues_adjusted)
25
50
75
100
Gene number
200
400
600
C
Turquoise module in HCC VS. yellow module in NAFLD
Cellular component
intracellular part
intracellular organelle part
intracellular
organelle part
intracellular membrane bounded organelle
membrane−bounded organelle
intracellular organelle
organelle
cytoplasmic part
membrane−enclosed lumen
NAFLD
HCC
−log10(pvalues_adjusted)
40
80
120
Gene number
500
1000
1500
2000
D
Brown module in HCC VS. yellow module in NAFLD
Cellular component
intracellular part
intracellular organelle part
intracellular
organelle part
intracellular membrane bounded organelle
membrane−bounded organelle
intracellular organelle
organelle
cytoplasmic part
membrane−enclosed lumen
NAFLD
HCC
−log10(pvalues_adjusted)
24
28
32
36
Gene number
300
400
500
600
