# Supplementary material for: The Gene Signature Associated with Hepatocellular Carcinoma in Patients with Nonalcoholic Fatty Liver Disease
Source: J Oncol. 2021 Apr 2;2021:6630535. doi: 10.1155/2021/6630535 (PMC8035011; doi:10.1155/2021/6630535)
Supplement: Supplementary Materials — Supplementary Table 1. The top 10 enriched biological processes of HANM. Supplementary Table 2. The enriched signaling pathways in the inflammatory response biological process. Supplementary Figure 1. WGCNA analysis of expression profiles of human NAFLD and healthy samples. Gene expression of human NAFLD and healthy samples was hierarchically clustered and dynamically cut into gene modules named by different colors. Gene modules with highly correlated eigengenes (Pearson's correlation coefficient > 0.75) were merged. Supplementary Figure 2. WGCNA analysis of expression profiles of human HCC samples. Gene expression of human HCC samples was hierarchically clustered and dynamically cut into gene modules named by different colors. Gene modules with highly correlated eigengenes (Pearson's correlation coefficient > 0.75) were merged. Supplementary Figure 3. Functional association between NAFLD and HCC. (a) Molecular function similarity between the turquoise module in HCC and the yellow module in NAFLD. (b) Molecular function similarity between the brown module in HCC and the yellow module in NAFLD. (c) The cellular component similarity between the turquoise module in HCC and the yellow module in NAFLD. (d) The cellular component similarity between the brown module in HCC and the yellow module in NAFLD. [file 6630535.f1.zip › 6630535.f1/Supplemental Table 1.docx]

Supplemental Table 1. The top 10 enriched biological processes of HANM

| GOID | #Hit | #Background | pvalues | go_terms |
| --- | --- | --- | --- | --- |
| GO:0006954 | 50 | 462 | 4.33E-20 | inflammatory response |
| GO:0009893 | 145 | 3174 | 8.3E-18 | positive regulation of metabolic process |
| GO:0051173 | 135 | 2852 | 1.01E-17 | positive regulation of nitrogen compound metabolic process |
| GO:0048519 | 193 | 4887 | 1.35E-17 | negative regulation of biological process |
| GO:0031325 | 138 | 2960 | 1.36E-17 | positive regulation of cellular metabolic process |
| GO:0010604 | 137 | 2945 | 2.29E-17 | positive regulation of macromolecule metabolic process |
| GO:0034097 | 65 | 897 | 1.08E-16 | response to cytokine |
| GO:0042981 | 87 | 1479 | 1.54E-16 | regulation of apoptotic process |
| GO:0043067 | 87 | 1494 | 2.83E-16 | regulation of programmed cell death |
| GO:0010941 | 91 | 1609 | 3.08E-16 | regulation of cell death |
